# Development of Type 1 Diabetes Mellitus in Nonobese Diabetic Mice Follows Changes in Thymocyte and Peripheral T Lymphocyte Transcriptional Activity

**DOI:** 10.1155/2011/158735

**Published:** 2011-06-15

**Authors:** Thais A. Fornari, Paula B. Donate, Claudia Macedo, Elza T. Sakamoto-Hojo, Eduardo A. Donadi, Geraldo A. Passos

**Affiliations:** ^1^Molecular Immunogenetics Group, Department of Genetics, Faculty of Medicine of Ribeirão Preto, University of São Paulo (USP), 14040-900, School Ribeirão Preto, SP, Brazil; ^2^Department of Biology, School of Philosophy, Science and Letters of Ribeirão Preto, USP, 14040-900 Ribeirão Preto, SP, Brazil; ^3^Department of Clinical Medicine, Faculty of Medicine of Ribeirão Preto, USP, 14040-900, Ribeirão Preto, SP, Brazil; ^4^Disciplines of Genetics and Molecular Biology, Department of Morphology (DMEF), School of Dentistry of Ribeirão Preto, USP, 14040-900 Ribeirão Preto, SP, Brazil

## Abstract

As early as one month of age, nonobese diabetic (NOD) mice feature pancreatic infiltration of autoreactive T lymphocytes, which destruct insulin-producing beta cells, producing autoimmune diabetes mellitus (T1D) within eight months. Thus, we hypothesized that during the development of T1D, the transcriptional modulation of immune reactivity genes may occur as thymocytes mature into peripheral T lymphocytes. The transcriptome of thymocytes and peripheral CD3^+^ T lymphocytes from prediabetic or diabetic mice analyzed through microarray hybridizations identified 2,771 differentially expressed genes. Hierarchical clustering grouped mice according to age/T1D onset and genes according to their transcription profiling. The transcriptional activity of thymocytes developing into peripheral T lymphocytes revealed sequential participation of genes involved with CD4^+^/CD8^+^ T-cell differentiation (Themis), tolerance induction by Tregs (Foxp3), and apoptosis (Fasl) soon after T-cell activation (IL4), while the emergence of T1D coincided with the expression of cytotoxicity (Crtam) and inflammatory response genes (Tlr) by peripheral T lymphocytes.

## 1. Introduction

Type 1 diabetes (T1D) is an autoimmune disease that results in the destruction of pancreatic insulin-producing beta cells [1, 2]. This destruction is a progressive process that occurs over five to eight months in the nonobese diabetic (NOD) mouse or several years in human patients [3]. The early stages of T1D pathogenesis are characterized by insulitis, an inflammation of the beta cells of the pancreas caused by lymphocyte infiltration. Nevertheless, the molecular genetics regulating the progress of beta cell failure and factors determining time of presentation of clinical diabetes are still poorly understood. 

The NOD mouse is an autoimmune mouse strain and is a primary animal model used to dissect the mechanisms of lack of immune tolerance and autoimmune T1D, which reflects at least a part of human T1D [4–6]. The most important genetic determinants in susceptibility to diabetes lie in the major histocompatibility complex (MHC). Within the MHC locus, the class II molecules DQ8 and DQ2 in humans and the mouse homologue I-Ag^7^ in the NOD mouse are thought to be particularly crucial [7]. In addition, many other genes have been identified that contribute to the development of diabetes in the NOD mouse [8].

In this murine strain, it is now clear that both the CD4^+^ and CD8^+^ subsets of T-cells play a role in the development of disease. Diabetes does not occur in the absence of CD4^+^ cells, as shown by studies using anti-CD4 antibodies [9] as well as in mice that lack CD4^+^ T-cells [10], mice that are deficient in CD8^+^ cells, either by anti-CD8 antibody injection into young mice [11], or mice in which few CD8^+^ T-cells develop because of a genetic lack of Beta-2 microglobulin [12–14]. These findings support the idea that T1D is a function of the action of autoreactive CD3^+^ T-cells that feature either a CD4^+^ or CD8^+^ phenotype. 

The BDC2.5 line, which derives from a CD4^+^ T-cell clone that is restricted by the NOD MHC class II A^g7^ molecule and specific for an unknown beta cell protein [15, 16], has been instrumental in the elucidation of several features of the immunoregulatory genes or cells that control the aggressively autoreactive T-cells in the periphery [17–20]. 

The differentiation into cytotoxic effector cells is the major function of CD8^+^ T-cells, which are able to recognize antigenic peptides in the context of MHC class I molecules. These peptides are produced through the endogenous antigen presenting pathways, though evidence suggests that exogenous antigens are also presented by MHC class I molecules [21, 22].

The thymus exerts an important role in controlling autoreactive T-cells. An extremely diverse repertoire of T-cells is generated through the random rearrangement of T-cell receptor (TCR) gene segments. This random process generates autoreactive T-cells that are eventually eliminated through negative selection, which occurs in the medullar compartment of the thymic stroma in close association with the medullary thymic epithelial cells (mTECs). The negative selection plays an essential role in preventing pathogenic autoimmune reactions and/or autoimmune diseases. 

The mTECs are essentially self-antigen-presenting cells. These cells express most of the parenchymal organs' self-antigens, a phenomenon that has been termed promiscuous gene expression (PGE) [23, 24]. Thymocytes are in close interaction with mTECs, establishing the thymic cross-talk. In fact, the self-antigens are coded from peripheral tissue antigen (PTA) genes. The translated PTAs are trimmed into peptides that are presented to thymocytes by means of the MHC. Dendritic cells also participate in the negative selection process after they have acquired PTA peptides from mTECs [23, 25–31].

Thymocyte clones that recognize self-peptide antigens during the cross-talk phase trigger a death gene expression cascade and die by apoptosis. Accordingly, the escaping autoreactive thymocytes from negative selection may cause severe aggressive reactions in the peripheral tissues and/or organs, provoking aggressive autoimmunity/autoimmune diseases. Thus, an imbalance in the central tolerance may have important consequences in the pathogenesis of autoimmune diseases, including T1D.

The central tolerance imbalance may explain, at least in part, the results of early studies using anti-CD3 antibodies; the results indicated that T1D in the NOD mouse is a T-cell-mediated disease [32].

We considered the following factors in our experiment: (1) peripheral T-cells represent the primary effectors in T1D autoreactivity in NOD mice; (2) the role played by autoreactive T-cells in the periphery may be a consequence of failure of the negative selection in the thymus; (3) the autoreactive phenotype of these cells may be a direct consequence of their transcriptional activity. Thus, our interest in this study was to analyze the transcriptome profile of thymocytes and peripheral CD3^+^ lymphocytes in the course of T1D in NOD mice to elucidate the sequential participation of genes associated with autoreactivity. 

## 2. Materials and Methods

### 2.1. Animals, Thymocytes and Peripheral T CD3^+^ Lymphocyte Isolation

Female NOD mice were born in specific pathogen-free (SPF) conditions at the CEMIB-UNICAMP animal facility of the University of Campinas, SP, Brazil and maintained in SPF mini-isolators in our laboratory at the University of São Paulo, Campus of Ribeirão Preto, SP, Brazil during the experiment. We studied prediabetic 1-month-old and diabetic 7-month-old animals. Diabetes was confirmed by blood glucose levels (≥250 mg glucose/dL) using the Accu-Chek Active kit (Roche Diagnostics Brazil, São Paulo, Brazil).

**Figure 1:**
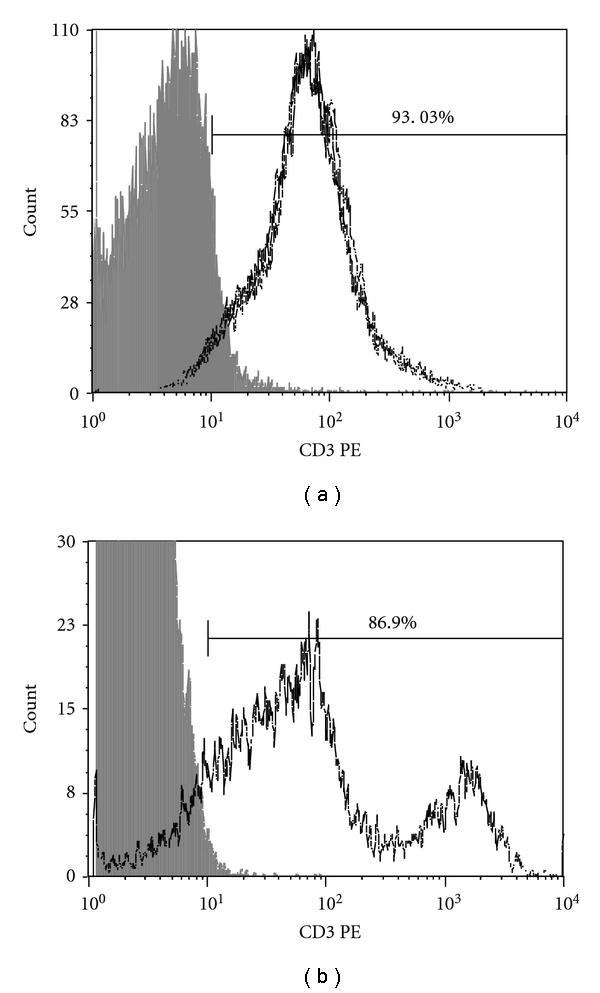
Fluorescent-activated cell sorting (FACS) analysis of thymocytes (approx. 93% purity) (a) and of peripheral CD3^+^ T lymphocytes (approx. 87% purity).

The thymi from 1-month-old animals were dissected and trimmed of fat and connective tissue in DMEM/F10 medium, and thymocytes were obtained by 2-3 passages of the thymic fragments throughout a 10-*μ*m mesh nylon membrane (Sefar Inc. Depew, NY, USA). Pelleted thymocytes were resuspended in phosphate-buffered saline (PBS). Fluorescent-activated cell sorting (FACS) analysis in a BD-FACScalibur flow cytometer with phycoerythrin-(PE-) labeled anti-CD3 antibody indicated that this procedure yielded approximately 93% purity of the thymocyte population (Figure 1(a)). These cells were then used for total RNA preparation.

The peripheral T CD3^+^ lymphocytes from 1-month-old prediabetic, 7-month-old prediabetic, or 7-month-old diabetic animals were isolated from spleens using magnetic beads for negative selection (Pan T-cell isolation kit, mouse, Miltenyi Biotec) according to the manufacturer's instructions. FACS analysis with PE-labeled anti-CD3 antibody indicated that this procedure yielded approximately 87% purity of the CD3^+^ T lymphocyte population (Figure 1(b)). These cells were then used for total RNA preparation. The animal experimental protocol was previously approved by the Commission for Ethics in Animal Research, Faculty of Medicine of Ribeirão Preto, USP, Brazil (Protocol no. 120/2008).

### 2.2. Total RNA Preparation

The total RNA was extracted from 1 × 10^7^ thymocytes or peripheral T CD3^+^ lymphocytes using a mirVana total RNA isolation kit (Ambion) according to the manufacturer's instructions. RNA preparations were confirmed to be free of proteins and phenol by UV spectrophotometry. The state of degradation was assessed by microfluidic electrophoresis using Agilent RNA Nano 6000 chips and an Agilent 2100 Bioanalyzer (Agilent Technologies, Santa Clara, CA, USA). Only RNA samples that were free of proteins and phenol and featured an RNA Integrity Number (RIN) ≥ 9.0 were used.

### 2.3. RNA Amplification, Labeling, Microarray Hybridization and, Data Analysis

Changes in gene expression were evaluated using the Agilent one-color (Cy3 fluorochrome) microarray-based gene expression platform according to the manufacturer's instructions. For hybridization onto whole mouse genome 4 × 44 K 60-mer oligonucleotide arrays (G4122F, Agilent Technologies, Palo Alto, CA, USA), 500 ng total RNA was used in the one-color Quick Amp labeling kit (Agilent Technologies, Santa Clara, CA, USA). Samples of complementary RNA (cRNA) were hybridized for 18 h at 42°C in a rotator oven and were then washed. The array slides were scanned using a DNA microarray scanner (Agilent Technologies), and the hybridization signals were extracted using the Agilent Feature Extraction software version 10.5. 

**Figure 2:**
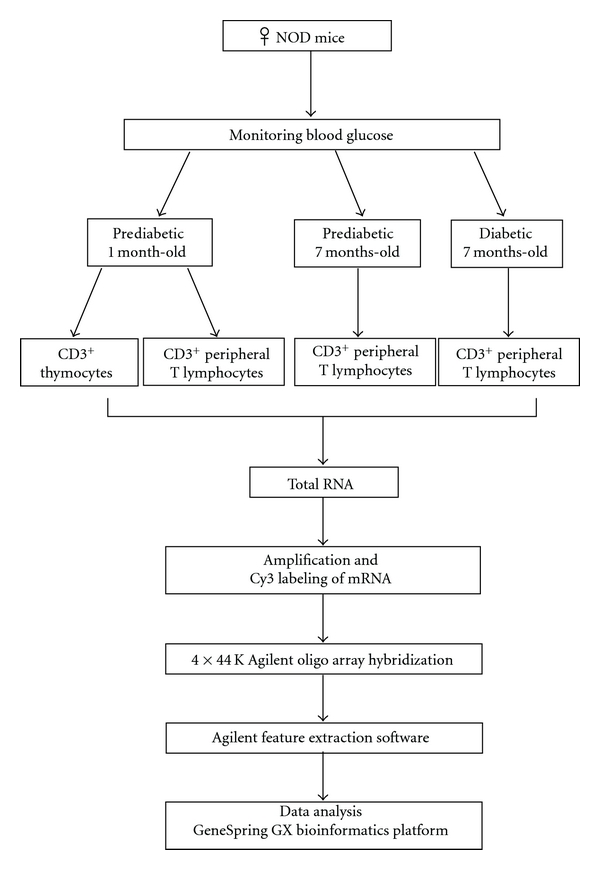
Experimental design of the work discriminating the biological samples used (animal groups and cell types), total RNA extraction, hybridizations, and microarray data analysis.

Gene expression profiles from independent preparations of thymocytes (from 1-month-old prediabetic mice) or CD3^+^ peripheral lymphocytes (from 1-month-old prediabetic, 7-month-old prediabetic or 7-month-old diabetic mice) were analyzed through comparisons of the microarray hybridizations of the respective samples.  Figure 2 depicts the experimental design for further comparison of the gene profiling. 

A complete file that provides all of the genes present in the microarray used in this study, as well as the experimental conditions, is available online at the MIAME public database [33], ArrayExpress accession E-MEXP 3047.

The microarray numerical quantitative data were normalized to the 75th percentile and were analyzed using the GeneSpring GX bioinformatics platform [34] according to the default instructions allowing hierarchical clustering of samples of mice or genes based on ANOVA statistical analysis (*P* < .01) with a fold change >2.0 and an uncentered Pearson correlation metrics [35]. The similarities and dissimilarities in gene expression are presented as dendrograms, in which the pattern and length of the branches reflect the relatedness of the samples or genes, and heat maps.

### 2.4. Gene Ontology

Microarray data analysis was used to identify gene expression based on combined information from the public databases DAVID [36] and SOURCE [37]. These databases show gene annotation enrichment analysis, functional annotation clustering, BioCarta and KEGG pathway mapping (DAVID), or microarray data and sequencing of cDNA clones from different organ/tissues (SOURCE), including GenBank accession number, chromosomal location and the molecular/biological function of each gene analyzed.

### 2.5. Oligonucleotide Primer Design and Quantitative Real-Time Polymerase Chain Reaction (qRT-PCR)

Microarray data were confirmed using qRT-PCR for the genes listed below that were differentially expressed between thymocytes and peripheral CD3^+^ T lymphocytes. The cDNA sequences of these genes were retrieved from the NCBI GenBank database (http://www.ncbi.nlm.nih.gov/genbank/GenbankSearch.html), and the Primer3 web tool (http://biotools.umassmed.edu/bioapps/primer3_www.cgi) was used to select pairs of oligonucleotide primers spanning an intron/exon junction and with consideration of the alternative transcripts. An optimal melting temperature of 60°C was standardized for all genes. The following forward and reverse sequences are given in the 5′ to 3′ orientation: Hypoxanthine phosphoribosyltransferase 1 (HPRT1, accession number NM_000194.2, GACCAGTCAACAGGGGACAT′ and CTGCATTGTTTTGCCAGTGT); Fas ligand (Fasl, accession number NM_000639, ACTCCGTGAGTTCACCAACC and GTGGGGGTTCCCTGTTAAAT); Toll-like receptor 3 (Tlr3, accession number BC068487, TTGTCTTCTGCACGAACCTG and CCCGTTCCCAACTTTGTAGA); Toll-like receptor 4 (Tlr4, accession number NM_138554, TCAGAACTTCAGTGGCTGGA CCTGGGGAAAAACTCTGGAT); Forkhead box P3 (Foxp3, accession number NM_014009, TCTTCGAGGAGCCAGAAGAG and GCTCCAGAGACTGCACCACT); Thymocyte selection associated (Themis, accession number NM_001164685, AAATGAAGCTCACCTTGCTCA and ATCCTGGCCACTTTCATCTG). HPRT1 was used as the constitutively expressed gene. Transcriptional expression levels were determined using a StepOne Real-Time PCR System (Applied Biosystems, USA). The ΔΔCT relative normalization method was used as described previously. We used the GraphPad Prism 5.00 tool (http://www.graphpad.com/prism/Prism.html) to run one-way or two-way ANOVAs with Bonferroni's correction statistics.

## 3. Results

Although the expression pattern remained unchanged between thymocytes and peripheral T lymphocytes from prediabetic and diabetic animals for the majority of the 44,000 sequences tested, which presented a control/test ratio *≈* 1.0 (Pearson correlation), 2,771 genes were found to be differentially expressed. Hierarchical clustering of the data allowed for the identification of clusters of upregulated (induced) and downregulated (repressed) genes. Changes in gene expression profile could be observed when comparing thymocytes from 1-month-old prediabetic mice with peripheral CD3^+^ lymphocytes from 1-month-old prediabetic, 7-month-old prediabetic, or 7-month-old diabetic mice to observe the modulated genes that coincided with the development of T1D. 

**Figure 3:**
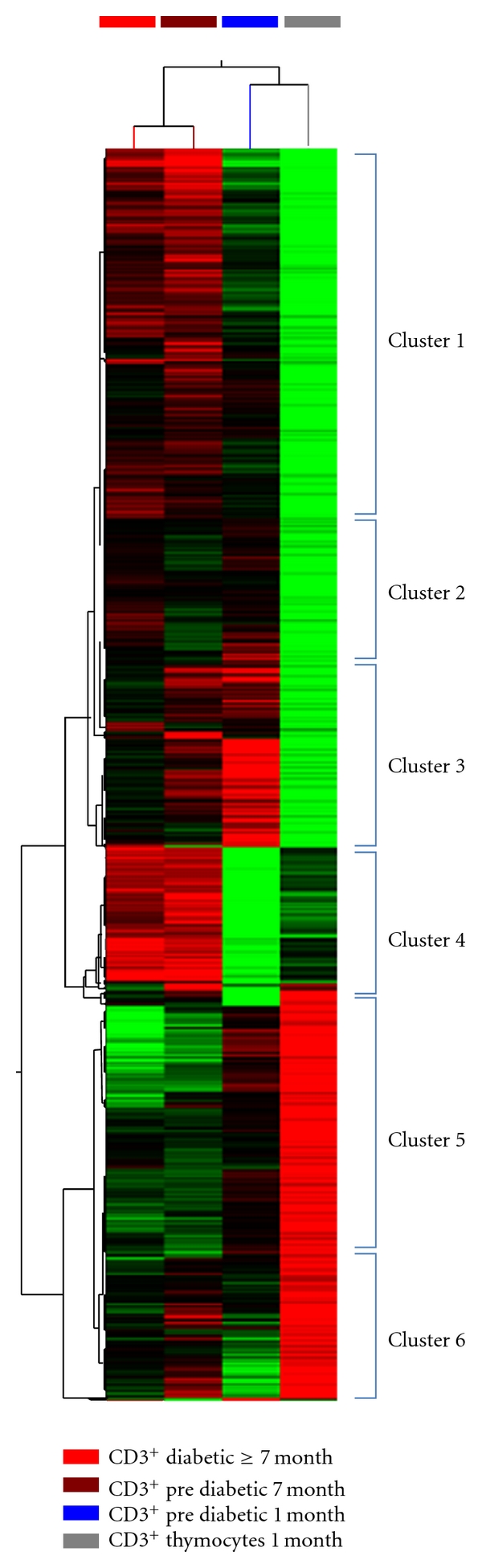
Hierarchical clustering of thymocytes and peripheral CD3^+^ lymphocytes of nonobese diabetic (NOD) mice based on microarray gene expression profiling. Dendrograms and heat maps were obtained using the Cluster-Tree View program within the GeneSpring GX (Agilent) platform. Red = upregulation, green = downregulation, and black = unmodulated (Pearson correlation metrics, 75 percentile). The 2,771 differentially expressed genes were divided in six clusters (clusters 1 to 6) according to their relative expression levels and ontology.

Hierarchical clustering analysis depicted in Figure 3 shows variability in the hybridization signatures between cell types. The upper horizontal dendrogram (cell samples) demonstrates that this variability could distinguish cells according to their developmental phase, namely, thymocytes (prediabetic animals) from peripheral CD3^+^ lymphocytes (prediabetic and diabetic animals). The left vertical dendrogram shows the genes that were differentially expressed (up- or downregulated) according to their respective biological function, and the genes were divided into six clusters (Figure 3 and Table 1). Clusters 1 to 4 contain genes downregulated in thymocytes and peripheral CD3^+^ lymphocytes from 1-month-old prediabetic animals and progressively upregulated in peripheral CD3^+^ lymphocytes from 7-month-old prediabetic and diabetic animals.

Cluster 1 comprises 767 differentially expressed genes, and the following genes were highlighted because they participate in immune processes potentially implicated in the pathogenesis of T1D: IL4, involved with T-cell activation, Crtam, involved in cytotoxicity and Tlr3, Tlr4, and Tlr6, involved in inflammatory response.

Cluster 2 comprises 340 differentially expressed genes, and the following genes were highlighted: Fasl, involved in the induction of apoptosis by extracellular signals and FoxP3, involved in CD25^+^ alpha-beta T-regulatory (Treg) cell differentiation and positive regulation of T-cell tolerance induction.

Cluster 3 comprises 435 differentially expressed genes, and the following genes were highlighted: Chemokine (C–C motif) ligand 4 gene (Ccl4) and Chemokine (C–C motif) receptor 2 gene (Ccr2), both involved in inflammatory response; CD48 antigen gene, involved in T-cell activation; Cytotoxic T-lymphocyte-associated protein 4 gene (Ctla4), involved in negative regulation of Treg cell differentiation; Interferon gamma gene (Ifng), involved in positive regulation of T-cell proliferation; Integrin alpha M gene (Itgam), involved in cell-cell adhesion.

Cluster 4 comprises 292 differentially expressed genes, and the following genes were highlighted: Integrin alpha X (Itgax) and Claudin 5 (Cldn5) gene, involved in cell-cell adhesion; Gap junction protein, alpha 5 (Gja5) gene, involved in cell-cell signaling; HNF1 homeobox 1 transcription factor, involved in maturity onset diabetes of the young and circadian regulation of transcription; and Gastrin (Gast) and Guanine nucleotide-binding protein 1 (Gna1) gene, both involved in protein signaling pathway. Interestingly, this cluster comprises various members of the olfactory receptor (Olfr) gene family, which is involved with cell signaling pathways.

Finally, clusters 5 and 6 comprise 610 and 327 genes, respectively, which feature a different expression pattern than those included in the previous clusters, that is, they were mainly upregulated in thymocytes from prediabetic mice and progressively downregulated in peripheral CD3^+^ lymphocytes from prediabetic and diabetic animals. These two clusters contain genes involved in CD4/CD8 cell differentiation (Themis) and V(D)J recombination (Rag1 and Rag2).

**Figure 4:**
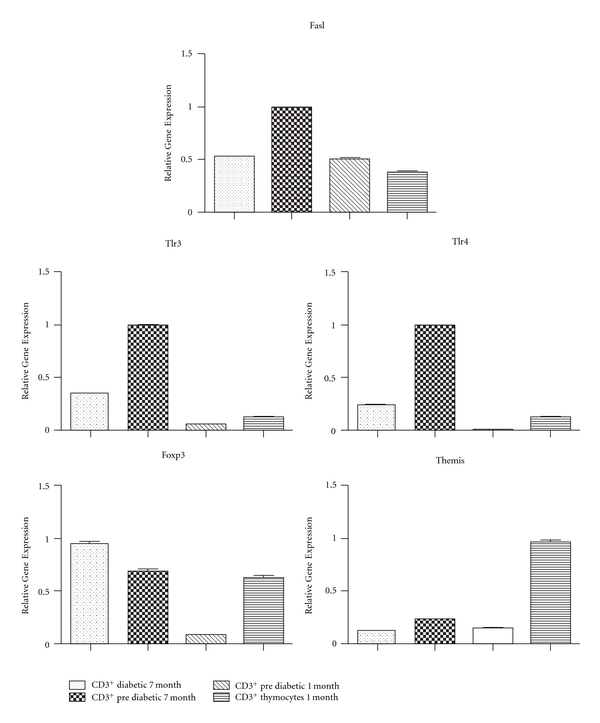
Confirmation of microarray data by qRT-PCR. The Fasl, Tlr3, and Tlr4 genes were downregulated in thymocytes from prediabetic animals, Foxp3 was upregulated in peripheral CD3^+^ lymphocytes from diabetic animals, and Themis was upregulated in thymocytes from prediabetic animals.

Using qRT-PCR, we assayed the expression levels of five differentially expressed genes (Fasl, Tlr3, Tlr4, Foxp3 and Themis) that are associated with thymocyte selection, T-cell differentiation, cell activation, or inflammation. The expression levels obtained with qRT-PCR method were comparable with microarrays, for example; the Fasl, Tlr3 and Tlr4 genes were downregulated in thymocytes from prediabetic animals, Foxp3 was upregulated in peripheral CD3^+^ lymphocytes from diabetic animals, and Themis was upregulated in thymocytes from prediabetic animals (Figure 4).

## 4. Discussion

In this study, we assessed the hypothesis that the transcriptional modulation of immune reactivity genes may occur during the development of T1D as thymocytes mature into peripheral T lymphocytes. Consequently, the emergence of T1D might follow a pattern of the transcriptional activity of these cells, sequentially featuring genes associated with a negative selection of thymocytes, T-cell maturation, differentiation, and autoreactivity.

As discussed in a previous study [38], diabetes in NOD mice is similar to T1D patients, and the progress of diabetes in these animals occurs in two stages. In the first stage, autoreactive CD8^+^ T lymphocytes infiltrate the pancreatic islet by 1 month after the birth. However, most pancreatic islets are preserved at this phase, and the animals are clinically healthy. Stage one can persist for months because the autoimmune attack is under control and is relatively nondestructive. In the second stage, most of pancreatic islets are destructed and animals are often diabetic.

Because these animals spontaneously develop autoimmune diabetes mellitus that is similar to human T1D, including the presence of pancreas-specific autoantibodies and autoreactive CD4^+^ or CD8^+^ T-cells and synteny to human chromosomal linkage groups associated with T1D, they are a classical model system for investigating autoimmune T1D and/or failure in the tolerance mechanisms [4, 6, 28]. Thus, we chose to employ the NOD mouse as a model system given the difficulty of easily testing this theory in humans.

To exclude the influence of genetic backgrounds of nonautoimmune mouse strains, we compared only groups of NOD mice in two distinct phases of autoimmunity, namely, prediabetic (1- or 7-month-old) and diabetic (≥7-month-old).

Moreover, by establishing transcriptome comparisons between cells from prediabetic and diabetic animals or between thymocytes and peripheral T lymphocytes, it was possible to find genes temporally regulated (because T1D emerges according to age) and regulated according to T-cell development. 

The procedures for isolation of thymocytes and peripheral CD3^+^ T lymphocytes used in this work yielded purities (approximately 93% for thymocytes and 85% for peripheral CD3^+^ T lymphocytes) that are comparable to automated cell sorting of T-cells. Moreover, the peripheral CD3^+^ T lymphocytes were isolated by negative separation, minimizing eventual cell activation by artifacts. 

Figure 3 (clusters 1 to 4) and Table 1 show the gene expression pattern observed in thymocytes and peripheral CD3^+^ lymphocytes from prediabetic 1-month-old animals. The gene expression in these cells could be considered to occur in the first stage of T1D, and this expression features the down-regulation of genes involved with the activation of the immune system/adaptive response, cell activation, NF-KappaB cascade, immune effector/immune response processes, inflammatory response, lymphocyte activation, the regulation of interferon-gamma production, the regulation of leukocyte-mediated cytotoxicity, apoptosis, cell communication, and signal transduction. Altogether, these biological processes are necessary for the development of lymphocyte differentiation and autoreactivity.

The thymocytes from prediabetic 1-month-old animals featured down-regulation of genes associated with several biological processes that were gradually upregulated in the peripheral CD3^+^ lymphocytes from prediabetic 1-month-old, prediabetic 7-month-old, and diabetic 7-month-old animals (Figure 3 and Table 1). Among these processes and their respective genes, we selected some of the most important to discuss because of their direct or indirect association with aggressive autoimmunity. 

The apoptosis process featured genes including Fas ligand (Fasl), TNF receptor-associated factor (Traf1 and Traf5), Traf3 interacting protein 2 (Traf3ip2), and Tumor necrosis factor (ligand) superfamily, member 8 (Tnfsf8 or TRAIL). 

Because apoptosis is the final stage of the negative selection of thymocytes in the thymus [29], the down-regulation of these genes may favor the survival of autoreactive thymocyte clones, including those that recognize pancreatic beta cell autoantigens. 

A role for TRAIL in T1D in NOD mice has been evidenced by its blockade with consequent exacerbation of the disease [39] and by its systemic delivery indicating that T1D can be prevented by TRAIL overexpression through an enhancement of the tissue inhibitor of the metalloproteinase-1 (TIMP-1) function [40]. The authors concluded that elevated TIMP-1 production inhibits the activity of matrix metalloproteinases, which may contribute to the suppression of the transmigration of diabetogenic T-cells into the pancreatic islets and protects pancreatic beta cells from cytokine-induced apoptosis.

The tolerance induction process featured Forkhead box P3 (Foxp3) gene, which was also downregulated in thymocytes from 1-month-old prediabetic animals. This gene is involved in tolerance induction via CD4^+^CD25^+^ T-regulatory cells (Tregs) [41–43], a process that directly activates any of the steps required for tolerance, a physiologic state in which the immune system does not react destructively against self-components. Thus, their deregulation may impair the Treg-mediated suppression of aggressive autoimmunity, favoring the survival of autoreactive thymocytes in the thymus.

The T-cell activation process featured several downregulated genes in thymocytes from 1-month-old prediabetic animals, from which we highlight Chemokine (C–C ligand motif) ligand 4 (Ccl4), Chemokine (C–C motif) receptor 2 (Ccr2), Cd48 antigen and Interferon, gamma (Ifng), and Interleukin 4 (IL4) genes. These genes were gradually upregulated in peripheral CD3+ lymphocytes from prediabetic to diabetic animals and may increase the activation of autoreactive T-cell clones in the periphery. 

Interestingly, the G-protein-coupled receptor protein signaling pathway and signal transduction processes shared several members of the olfactory gene family (Olfr), which is involved in sensory perception of smell through receptor and signal transducer activity. The two pathways also shared bone morphogenetic protein 8a (Bmp8a) gene, which is involved in growth and ossification. These genes were downregulated in thymocytes from 1-month-old prediabetic animals and were gradually upregulated in peripheral CD3^+^ lymphocytes from prediabetic to diabetic animals. Despite their disparate biological processes in the context of T1D, these genes may play a role in the receptor-mediated signal transduction activity of peripheral T-cells in diabetic animals. 

Dissimilar from the pattern of genes related to the processes discussed above, the T-cell receptor V(D)J recombination and CD4^+^/CD8^+^ T-cell differentiation featured genes were upregulated in thymocytes and CD3^+^ peripheral T lymphocytes from prediabetic 1-month-old animals and were gradually downregulated in peripheral CD3+ lymphocytes from prediabetic and diabetic 7-month-old animals. 

Among these genes, we highlight the recombination activating genes (Rag-1 and Rag-2) that code the recombinase catalytic complex involved in the recognition of recombination signal sequences (RSS) within the T-cell receptor loci (TCR alpha, beta, gamma or delta), cutting and recombining DNA during aleatory generation of TCR diversity. The generation of TCR diversity implies the production of T-cell clones directed to foreign antigens and also autoreactive clones. Autoreactive clones are normally eliminated by apoptosis throughout the negative selection process. 

Also, Adenosine deaminase (Ada) gene, which is involved in a process that increases the frequency of T-cell differentiation in the thymus, and Cytotoxic T-lymphocyte-associated protein 4 (Ctla4) gene, which in contrast to Ada gene, is involved in the negative regulation of T-cell proliferation, were both identified.

Finally, we found the Thymocyte selection associated (Themis) gene, which is expressed in the thymus and to a lesser extent in the spleen but is not detectable in nonlymphoid tissues. This gene is highly expressed in thymocytes between the pre-T-cell antigen receptor (pre-TCR) and positive-selection checkpoints and is expressed at a low level in mature T-cells (at the protein level). Themis is also implicated in the control of T-helper CD4+/cytotoxic CD8+ cell fate. Moreover, the CD4 as well as CD8a antigen genes, which define the respective T-helper CD4^+^ or T cytotoxic CD8+ phenotypes, were also found. 

 During their permanence within the thymus, thymocytes activate a developmentally complex mechanism because of close contact with thymic stroma. The medullary thymic epithelial cells (mTECs), which form the stromal medullar compartment, present peripheral tissue antigens (PTAs) to thymocytes to eliminate autoreactive clones by inducing apoptosis (negative selection), leading to tolerance induction [23, 24]. 

Failure in the expression of specific PTA genes in the thymic stroma is strongly associated with aggressive autoimmunity as recently observed during the development of T1D in NOD mice [38] or collagen-induced arthritis in DBA-1/J mice [44]. This indeed led to survival of autoreactive thymocyte clones that once in the periphery, mediate autoimmune attack of target structures such as pancreatic beta cells.

To better understand the control of transcriptional activity of thymocytes specifically associated with the induction of tolerance and negative selection within the thymus or cytotoxicity and inflammatory response in peripheral T lymphocytes, we propose further experiments to evaluate the participation of microRNAs (miRNAs) that once dysregulated might be associated with aggressive autoimmunity of T1D.

## 5. Conclusion

In this study, we were able to construct a transcriptional profile of T-cell development comparing thymocytes with peripheral CD3+ lymphocytes in the context of the emergence of T1D. The sequential participation of genes involved with the main steps of T-cell development such as the generation of TCR diversity, CD4^+^/CD8^+^ cell fate, apoptosis, and negative selection demonstrated that the T1D autoimmune phenotype in NOD mice runs in parallel with transcriptome changes of T-cells. The results obtained confirm our initial hypothesis.

## Figures and Tables

**Table 1:** Clusters of the differentially expressed genes and their ontology.

Cluster	Biological process	Genes
	Activation of immune system	Cd55 Daf2 Fcer1g Klre1 Klrk1 Lax1 Lyn Malt1 Masp2 Plcg2 Tlr3 Tlr4 Tlr6 Unc93b1
	Adaptive immune response	Bcl3 Cd55 Cd74 Daf2 Fcer1g Fcgr3 Icam1 Icosl Lilrb3 Masp2 Pou2f2 Slc11a1 Tlr6
	Cell activation	Bank1 Bcl11a Bcl3 Btk Casp1 Cd74 Clcf1 Cplx2 Cxcr5 Elf4 Entpd1 Fcer1g Fcgr3 Fyb Gapt Gpr183 H2-M3 Hdac9 Hhex Icosl Il4 Irf1 Irf4 Itgax Klre1 Klrk1 Lax1 Lbp Lilrb3 Lyn Malt1 Plcg2 Pou2f2 Slc11a1 Tlr3 Tlr4 Tlr6 Vwf
	Defense response	Alox5 Bcl3 Btk Ccl19 Ccl5 Ccr2 Ccr5 Cd163 Cd180 Cd36 Cd55 Cd74 Chst2 Ciita Clec4a2 Clec4d Cnr2 Daf2 Ddx58 Fcer1g Fcgr3 H2-K1 H2-M3 Hdac9 Il18rap Il1b Irf8 Lbp Lta Ly86 Lyn Malt1 Masp2 Mefv Ncf1 Neurod2 Pglyrp1 Prg2 Samhd1 Slc11a1 Tirap Tlr3 Tlr4 Tlr6
	I-kappa8 kinase/NF-kappa8 cascade	Btk Irak2 Malt1 Rel Tirap Tlr4
1	Immune effector process	Bcl3 Btk Cd55 Cd74 Cplx2 Daf2 Fcer1g Fcgr3 Icam1 Icosl Lax1 Lbp Lilrb3 Lyn Masp2 Ncf1 Pou2f2 Slc11a1
	Immune response	Bcl3 Btk Ccl19 Ccl5 Ccl9 Ccr2 Cd180 Cd55 Cd74 Ciita Clec4a2 Clec4d Cplx2 Daf2 Ddx58 Enpp1 Fasl Fcer1g Fcgr3 Fcgrt Gpr183 H2-D1 H2-K1 H2-M3 H2-Q10 H2-Q2 H2-Q7 H2-Q8 H2-T23 Hfe Icam1 Icosl Igj Il18rap Il1b Il4 Irf8 Irf8 Lax1 Lbp Lilrb3 Lta Ltb Ly86 Lyn Malt1 Masp2 Ncf1 Oas1b Pglyrp1 Plcg2 Pou2f2 Prg2 Samhd1 Slc11a1 Tirap Tlr3 Tlr4 Tlr6
	Immune response-activating signal transduction	Fcer1g Klre1 Klrk1 Lax1 Lyn Malt1 Plcg2 Tlr3 Tlr4 Tlr6 Unc93b1
	Immune system process	Bank1 Bcl11a Bcl3 Btk Casp1 Ccl19 Ccl5 Ccl9 Ccr2 Cd180 Cd300lf Cd55 Cd74 Ciita Clcf1 Clec4a2 Clec4d Cplx2 Crkl Csf1 Csf3r Cxcr5 Daf2 Ddx58 Dnase2a Elf4 Enpp1 Fasl Fcer1g Fcgr3 Fcgrt Fyb Gapt Gpr183 H2-D1 H2-K1 H2-M3 H2-Q10 H2-Q2 H2-Q7 H2-Q8 H2-T23 Hdac9 Hfe Hhex Icam1 Icosl Igj Il18rap Il1b Il4 Irf1 Irf4 Irf8 Itgax Klre1 Klrk1 Lax1 Lbp Lilrb3 Lta Ltb Ly86 Lyn Malt1 Masp2 Myo1e Ncf1 Oas1b Pglyrp1 Plcg2 Pou2f2 Prg2 Samhd1 Slc11a1 Terc Tirap Tlr3 Tlr4 Tlr6 Tnfrsf13c Unc93b1
	Inflammatory response	Alox5 Btk Ccl19 Ccl5 Ccr2 Cd163 Cd180 Cd55 Chst2 Cnr2 Daf2 Fcgr3 Hdac9 Il1b Lbp Lta Ly86 Lyn Masp2 Mefv Ncf1 Slc11a1 Tirap Tlr3 Tlr4 Tlr6
	Lymphocyte activation	Bank1 Bcl11a Bcl3 Cd74 Clcf1 Cxcr5 Elf4 Gapt Gpr183 H2-M3 Hdac9 Hhex Icosl Il4 Irf1 Itgax Klre1 Klrk1 Lax1 Malt1 Plcg2 Pou2f2 Slc11a1
	Lymphocyte activation during immune response	Bcl3 Gpr183 H2-M3 Plcg2 Slc11a1
	Lymphocyte mediated immunity	Bcl3 Cd55 Cd74 Daf2 Fcer1g Fcgr3 Icam1 Icosl Lilrb3 Masp2 Pou2f2 Slc11a1
	Positive regulation of interferon-gamma production	Bcl3 H2-M3 Irf8 Klre1 Klrk1 Lta Slc11a1 Tlr4
	Regulation of defense response	Adrb2 Anxa1 Cadm1 Ccl5 Ccr5 Cnr2 Crtam Fcer1g Fcgr3 H2-Bl H2-M3 Klrb1b Klre1 Klrk1 Lta Nt5e Tgm2 Tlr3 Tlr4 Tlr6 Unc93b1
	Regulation of immune effector process	Cadm1 Crtam Fcer1g Fcgr3 H2-Bl H2-K1 H2-M3 Hmox1 Klrb1b Klre1 Klrk1 Lta
	Regulation of immune response	Btla Cadm1 Cd55 Crtam Daf2 Fcer1g Fcgr3 H2-Bl H2-K1 H2-M3 Il4 Klrb1b Klre1 Klrk1 Lax1 Lta Lyn Malt1 Masp2 Plcg2 Slc11a1 Tlr3 Tlr4 Tlr6 Tnfrsf13c Unc93b1
	Regulation of inflammatory response	Adrb2 Anxa1 Ccl5 Cnr2 Fcer1g Fcgr3 Lta Nt5e Tgm2 Tlr4
	Regulation of leucocyte mediated cytotoxicity	Cadm1 Crtam H2-Bl H2-K1 H2-M3 Klrb1b Klre1 Klrk1
	Apoptosis	Actc1 Bag3 Dapl1 Dedd2 Fasl Gramd4 Hipk2 Nfkb1 Nod1 Pea15a Pim2 Pmaip1 Psen2 Ripk1 Sgk1 Sgms1 Shisa5 Tmem173 Traf1 Traf3ip2 Traf5
	Immune response	B2m Ccl3 Ccl5 Cxcl9 Eomes Fasl Foxp3 H2-Q10 Il18r1 Il1rl1 Il7r Irgm1 Myo1f Pf4 Psen2 Tgtp1 Tlr1 Tmem173 Tnfaip8l2 Tnfsf8 Traf3ip2

2	Immune system process	B2m Ccl3 Ccl5 Cxcl9 Eomes Fasl Flt3l Foxp3 Gimap5 H2-Q10 Il18r1 Il1rl1 Il2rb Il7r Irf1 Irgm1 Jak3 Myo1f Nfkb1 P2rx7 Pf4 Pik3cd Psen2 Slamf1 Tgfbr2 Tgtp1 Tlr1 Tmem173 Tnfaip8l2 Tnfsf8 Traf3ip2
	Regulation of I-kappaB kinase/NF-kappaB cascade	Card6 Il1rl1 Nod1 Pim2 Tgm2
	Regulation of signal transduction	Arhgef12 Arhgef3 Arrb1 Arrb2 Axin2 Card6 Cd44 Fasl Furin Il1rl1 Nod1 P2rx7 Pim2 Psen2 Rasa3 Rasgrp2 Rgs11 Runx2 S1pr1 Smad7 Socs3 Spry2 Tgm2 Zeb2

3	Immune system process	Add2 Ahsp Ank1 Ccl4 Ccr2 Cd48 Cebpa Ctla4 Ctse Elane Epas1 Epb4.2 Gimap5 Gm5077 Id2 Ifng Il1r1 Il1rl1 Il1rl2 Itgam Junb Klf1 Klf11 Mpo Plscr1 Polr3c Samhd1 Spna1 Tal1 Tgtp1 Trim10 Txnrd2 Zbtb32

4	Cell communication	Atg16l1 Bmp8a Cacna1c Cblc Chat Cldn5 Cxcr7 Drd5 Erbb3 Gad1 Gad2 Gast Gja5 Gnal Gpr12 Gpr173 Gpr82 Grp Grpr Hnf1b Itgax Kcnk2 Lin7a Mrgprb2 Ngfr Olfr1010 Olfr1022 Olfr1048 Olfr107 Olfr1090 Olfr1115 Olfr1161 Olfr128 Olfr1377 Olfr1384 Olfr1388 Olfr1459 Olfr1462 Olfr1469 Olfr1495 Olfr304 Olfr33 Olfr350 Olfr365 Olfr516 Olfr523 Olfr556 Olfr606 Olfr62 Olfr651 Olfr68 Olfr684 Olfr724 Olfr768 Olfr770 Olfr784 Olfr790 Olfr796 Olfr845 Olfr889 Olfr904 Olfr924 Olfr974 Park2 Pdx1 Pik3c2g Plat Pth Rab3b Rab3c Slc1a2 Slc6a4 Syn2 Taar4 Tacr1 Upk1a Vmn2r26 Vmn2r81 Xcr1
	G-protein coupled receptor protein signaling pathway	Cxcr7 Drd5 Gast Gnal Gpr12 Gpr173 Gpr82 Grp Grpr Kcnk2 Mrgprb2 Olfr1010 Olfr1022 Olfr1048 Olfr107 Olfr1090 Olfr1115 Olfr1161 Olfr128 Olfr1377 Olfr1384 Olfr1388 Olfr1459 Olfr1462 Olfr1469 Olfr1495 Olfr304 Olfr33 Olfr350 Olfr365 Olfr516 Olfr523 Olfr556 Olfr606 Olfr62 Olfr651 Olfr68 Olfr684 Olfr724 Olfr768 Olfr770 Olfr784 Olfr790 Olfr796 Olfr845 Olfr889 Olfr904 Olfr924 Olfr974 Pth Taar4 Tacr1 Vmn2r26 Vmn2r81 Xcr1
	Signal transduction	Bmp8a Cblc Cxcr7 Drd5 Erbb3 Gast Gnal Gpr12 Gpr173 Gpr82 Grp Grpr Itgax Kcnk2 Mrgprb2 Ngfr Olfr1010 Olfr1022 Olfr1048 Olfr107 Olfr1090 Olfr1115 Olfr1161 Olfr128 Olfr1377 Olfr1384 Olfr1388 Olfr1459 Olfr1462 Olfr1469 Olfr1495 Olfr304 Olfr33 Olfr350 Olfr365 Olfr516 Olfr523 Olfr556 Olfr606 Olfr62 Olfr651 Olfr68 Olfr684 Olfr724 Olfr768 Olfr770 Olfr784 Olfr790 Olfr796 Olfr845 Olfr889 Olfr904 Olfr924 Olfr974 Pdx1 Pik3c2g Plat Pth Rab3b Rab3c Taar4 Tacr1 Upk1a Vmn2r26 Vmn2r81 Xcr1
	Apoptosis, apoptosis regulation	Alms1 Bcl2l1 Birc5 Bub1 Bub1b Casp6 Ckap2 Cul7 E2f1 E2f2 Egln3 Epha2 Fas Ift57 Krt18 Krt8 Lig4 Phlda1 Rad21 Rtn3 Stk3 Tfdp1 Tia1 Tpx2 Traf4 Trp53inp1 Vdac1
	Cell activation	Ada Bcl11b Ccnd3 Cd4 Cd8a Cxcl12 Fas Hdac7 Hells Lig4 Ly6d Msh6 Patz1 Rag1 Rorc Satb1 Sox4 Themis

5	Cell cycle	1190002H23Rik Anapc5 Anln Aspm Aurka B230120H23Rik Birc5 Bub1 Bub1b C79407 Casc5 Ccdc99 Ccna2 Ccnb1 Ccnb2 Ccnd3 Ccne2 Ccnf Ccng2 Cdc20 Cdc25a Cdc25c Cdc45 Cdca2 Cdca3 Cdca5 Cdk1 Cdkn1a Cdkn2c Cdkn3 Cenpe Cenpj Cep55 Chek1 Ckap2 Ckap5 Cul7 E2f1 E2f2 E2f3 E2f7 Ercc6l Esco2 Espl1 F630043A04Rik Fam33a Fbxo5 Gas2l3 Gsg2 H2afx Haus2 Hells Kif11 Kif2c Kifc1 Lig4 Mad1l1 Mki67 Myb Ncapd2 Ncaph Ndc80 Nde1 Nek2 Nsl1 Nuf2 Nusap1 Pard6g Phgdh Prc1 Psrc1 Pttg1 Rad21 Rad51c Rbbp4 Rcc1 Sgol1 Sgol2 Skp2 Spag5 Spc25 Stmn1 Tacc2 Tacc3 Tfdp1 Tpx2 Trp53inp1 Tubb5 Tubg1 Ube2c Uhrf1 Wee1
	Cell proliferation	Alms1 Aspm Bcl2l1 Ccnd3 Cxcl12 Gins1 Hells Hmgb1 Lig4 Lipa Mki67 Ncapg2 Nde1 Satb1 Tacc2 Tacc3 Uhrf1 Vegfa
	Lymphocyte activation	Ada Bcl11b Ccnd3 Cd4 Cd8a Cxcl12 Faz Hdac7 Hells Lig4 Ly6d Msh6 Patz1 Rag1 Rorc Satb1 Sox4 Themis
	Lymphocyte differentiation	Ada Bcl11b Cd4 Cd8a Fas Hdac7 Hells Lig4 Ly6d Patz1 Rag1 Rorc Satb1 Sox4 Themis
	T-cell activation	Bcl11b Ccnd3 Cd4 Cd8a Cxcl12 Fas Lig4 Patz1 Rag1 Rorc Satb1 Sox4 Themis
	T-cell differentiation	Bcl11b Cd4 Cd8a Fas Lig4 Patz1 Rag1 Rorc Satb1 Sox4 Themis
	V(D)J recombination	Bcl11b Lig4 Rag1 Xrcc6
	CD8-positive, alpha-beta T-cell differentiation	Pax1 Satb1
	Cell differentiation	Acan Bcl11b Bcl2l1 Bcl6 Bmp7 Cby1 Cdkn1c Cux1 Cxcl12 Dyrk1b Ephb2 Gjc1 Gpc2 Hdac2 Id3 Ift81 Igfbp3 Lhx2 Lig4 Morc1 Msi2 Myh10 Notch1 Notch3 Ntn1 Paqr5 Pax1 Pias2 Pitx2 Ptprf Rag1 Rag2 Runx1 Satb1 Sox11 Spata6 Spo11 Stra8 Tbata Thy1 Whrn

6	Cell-cell adhesion	Acan Arvcf Jup Lmo4 Mcam Ncam1 Ntn1 Ptprf Pvrl3 Vangl2
	Lymphocyte differentiation	Bcl11b Bcl6 Lig4 Pax1 Rag1 Rag2 Satb1
	Somatic diversification and recombination of T-cell receptor genes	Bcl11b Lig4
	T-cell activation	Bcl11b Cxcl12 Lig4 Pax1 Rag1 Rag2 Satb1 Sla2
	T-cell differentiation	Bcl11b Lig4 Pax1 Rag1 Rag2 Satb1
	T-cell receptor V(D)J recombination	Bcl11b Lig4
	V(D)J recombination	Bcl11b Lig4 Rag1 Rag2
